# Predictive value of systemic inflammatory response index for coronary artery disease and angiographic severity: a single-center retrospective study

**DOI:** 10.3389/fcvm.2026.1871311

**Published:** 2026-07-29

**Authors:** Yizhe Wang, Jinxiang Huang, Peng Zheng, Jiaqi Chen, Maoxiang Jin, Puyu Zhou, Qiang Zheng, Lianpin Wu

**Affiliations:** 1 Department of Cardiology, The Second Affiliated Hospital and Yuying Children’s Hospital of Wenzhou Medical University, Wenzhou, Zhejiang, China; 2 Zhejiang and Ireland International Joint Laboratory for Precise Diagnosis and Treatment of Heart Valve Disease, The Second Affiliated Hospital of Wenzhou Medical University, Wenzhou, China; 3 Key Laboratory of Panvascular Diseases of Wenzhou, The Second Affiliated Hospital and Yuying Children’s Hospital of Wenzhou Medical University, Wenzhou, Zhejiang, China

**Keywords:** coronary artery disease, Gensini score, inflammation, retrospective study, risk prediction, systemic inflammatory response index

## Abstract

**Background:**

Systemic inflammation plays a pivotal role in coronary artery disease (CAD). Systemic inflammatory response index (SIRI), a composite biomarker derived from neutrophil, monocyte, and lymphocyte counts, has been poorly studied for its association with angiographic CAD severity in East Asians. This study aimed to explore the relationship between SIRI and the presence and angiographic severity of CAD, and to develop a predictive model for risk stratification in a southeastern Chinese cohort.

**Methods:**

We retrospectively enrolled adult inpatients who underwent coronary angiography in Wenzhou, China, between June 2023 and March 2024. CAD was defined as ≥50% stenosis in at least one major epicardial coronary artery. Baseline clinical, laboratory, echocardiographic, and angiographic data were collected. SIRI and other inflammation-based indices were calculated from admission blood counts. Multivariable logistic regression was used to identify independent CAD predictors, and the final CAD model was evaluated using analysis of receiver-operating characteristic curves, internal bootstrap validation, calibration assessment, decision curve analysis, added predictive value analysis, stability testing, and subgroup analyses.

**Results:**

Among 394 patients, SIRI was higher in patients with CAD and increased across CAD phenotypes, with the highest values in the myocardial infarction groups. SIRI showed the highest AUC among the evaluated inflammatory indices for CAD discrimination. Albumin, left ventricular ejection fraction (LVEF), and SIRI remained independently associated with CAD. The albumin + LVEF + SIRI model showed moderate discrimination for angiographic CAD (AUC = 0.740, 95% CI: 0.682–0.797), improved the albumin + LVEF model (delta AUC = 0.045; DeLong *p* = 0.024), showed acceptable internal calibration, and remained stable when SIRI was modeled as untransformed, log_10_-transformed, or quartile-based values. A low-costnomogram (AUC = 0.74) showed favorable internal calibration and parallel performance for angiographic severity (AUC = 0.719).

**Conclusions:**

SIRI was independently associated with angiographic CAD and disease severity in this cohort. A predictive model integrating SIRI, albumin, and LVEF showed moderate discrimination and should be considered a preliminary, internally validated, hypothesis-generating risk-stratification framework rather than a clinically deployable decision-making tool.

## 1 Introduction

Data collected by the World Health Organization (WHO) indicate that ischemic heart disease (IHD, also called coronary artery disease, CAD) accounts for approximately 13% of all deaths worldwide (1). According to a 2025 Global Burden of Disease special report, CADs were responsible for an estimated 19.2 million deaths in 2023, marking a significant increase from 13.1 million in 1990 even under the control of conventional risk factors and the optimization of lifestyle interventions (2). Beyond conventional metabolic and hemodynamic risk factors, chronic vascular inflammation is recognized as a key contributor to atherosclerotic plaque initiation, progression, and destabilization (3). Low-cost inflammatory biomarkers derived from routine blood tests may therefore provide useful complementary information in patients undergoing coronary evaluation, particularly in clinical settings where rapid and broadly available indicators are needed.

The systemic inflammatory response index (SIRI), calculated as neutrophil count × monocyte count/lymphocyte count, integrates three leukocyte populations involved in atherosclerosis-related inflammation (4). Emerging evidence indicates that composite indices incorporating leukocyte subtypes, such as SIRI, enable better characterization of systemic inflammation and improve cardiovascular risk prediction (5). Although a panel of hematological inflammatory indices including NLR, PLR, MLR and SII have also been widely applied in CAD-related studies, their predictive performance varies greatly across distinct populations and clinical endpoints. For young patients with CAD, SIRI presents superior diagnostic efficacy for acute coronary syndrome (ACS) and coronary lesion severity compared with NLR, MLR, PLR and SII (6). In elderly patients with chronic coronary syndrome, SIRI also acts as a powerful independent predictor of ACS progression, with a much higher odds ratio than PLR and SII (7). Additionally, a previous large population-based study using the US National Health and Nutrition Examination Survey (NHANES) cohort demonstrated that PLR and SII failed to independently predict CAD after adjusting for confounders (8). Several studies have reported associations between SIRI and CAD occurrence, coronary lesion burden, acute coronary syndrome, or adverse cardiovascular outcomes. However, the relative performance of SIRI compared with other inflammatory indices varies across populations and clinical endpoints, and its integration with routinely available nutritional and cardiac functional indicators has not been sufficiently explored. Besides, serum albumin may reflect nutritional reserve, hepatic synthetic capacity, and a negative acute-phase response, whereas left ventricular ejection fraction (LVEF) reflects the functional consequence of ischemia, myocardial injury, and ventricular remodeling. These complementary domains provide a rationale for testing an inflammation-albumin-cardiac function framework. The southeastern Chinese population may have region-specific dietary, lifestyle, body habitus, and cardiometabolic characteristics that influence CAD risk patterns and the relative importance of inflammatory markers. Given the relatively low metabolic burden in this cohort, inflammatory factors may exert a relatively prominent role in CAD development, which makes SIRI worthy of exploration for risk stratification. This study investigated the association between admission SIRI and CAD presence, angiographic severity assessed by the Gensini score, and CAD phenotypes. It further assessed whether combining SIRI with albumin and LVEF provides a concise exploratory model for preliminary CAD risk stratification and could serve as a practical tool for CAD risk stratification in this regional population.

## 2 Materials and methods

### 2.1 Study design

This is a single-center, retrospective observational cohort study.

Study objectives: To explore the association between admission SIRI and the presence of CAD; to screen independent influencing factors of CAD; and to construct a predictive model for CAD risk evaluation.

Study population: Patients were eligible if they were aged 18 years or older, had complete inpatient medical records, and had angiographic confirmation of either obstructive CAD or non-obstructive coronary findings.

Primary exposure variable: Systemic inflammatory response index (SIRI).

Primary outcome indicators: The diagnosis of CAD (binary outcome).

### 2.2 Study population

**Figure 1:**
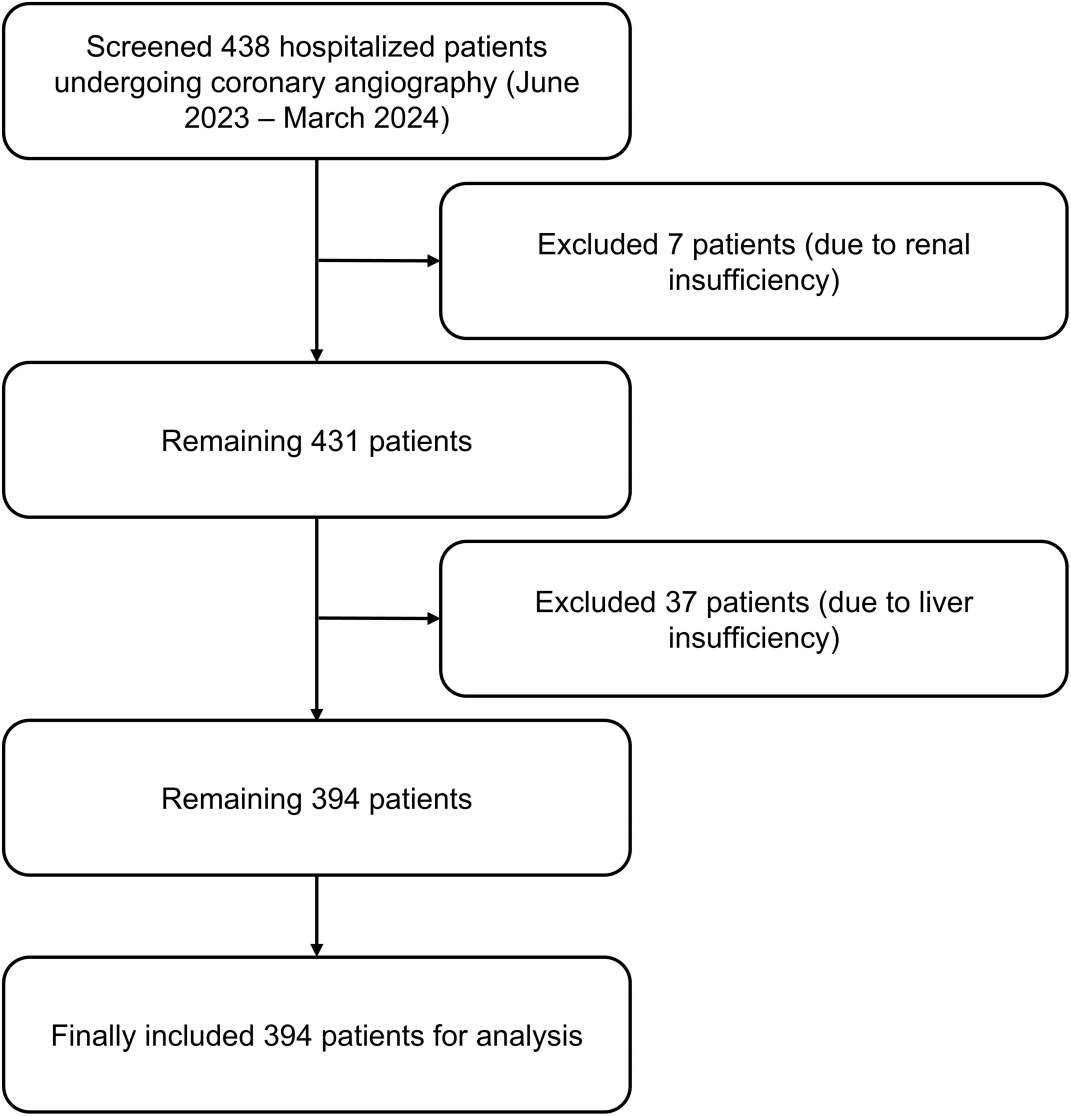
Flowchart of patient screening and enrollment. A total of 438 hospitalized adult patients who underwent coronary angiography between June 2023 and March 2024 were screened. After exclusion of 7 patients with renal insufficiency and 37 patients with hepatic dysfunction, 394 patients were included in the final analysis. These exclusion criteria were applied to reduce potential confounding related to systemic inflammation, blood cell profiles, and albumin metabolism.

This single-center retrospective observational study included consecutive adult inpatients who underwent coronary angiography at the Second Affiliated Hospital of Wenzhou Medical University from June 2023 to March 2024. Patients were eligible if they were aged 18 years or older, had complete inpatient medical records, and had angiographic confirmation of either obstructive CAD or non-obstructive coronary findings. Patients with renal insufficiency, defined as serum creatinine ≥133 µmol/L or clinically diagnosed renal dysfunction, were excluded because renal dysfunction may alter blood cell profiles, systemic inflammatory status, and albumin metabolism. Patients with hepatic dysfunction, defined as transaminase levels >3 times the upper limit of normal or a history of chronic liver disease, were also excluded because liver disease may influence hematopoiesis, immune cell distribution, hepatic synthetic function, and albumin level. After excluding seven patients with renal insufficiency and 37 patients with hepatic dysfunction, 394 patients were included in the final analysis (Figure 1). All clinical data, laboratory results, echocardiographic parameters, and angiographic reports were extracted from standardized electronic medical records and double-checked by two investigators to reduce data entry errors. The study protocol was approved by the hospital's ethics committee (Approval No.: 2024-K-078-01), and the requirement for informed consent was waived due to the retrospective design, in accordance with the Declaration of Helsinki.

### 2.3 Clinical data and definitions

Baseline clinical data, including demographics, medical history, vital signs, laboratory results, echocardiographic parameters, and medication use, were collected from electronic medical records. Hypertension, diabetes mellitus, dyslipidemia, and CAD was defined as ≥50% stenosis in at least one major epicardial coronary artery on coronary angiography according to current international guidelines (9–11). Coronary angiography was performed using radial or femoral artery access. As outlined in the 2021 ACC/AHA/SCAI Guideline for Coronary Artery Revascularization, this is the preferred method to assess stenosis in CAD (12). Coronary angiography was performed through radial or femoral artery access according to routine clinical practice. Angiograms were evaluated by experienced cardiologists blinded to laboratory results. Angiographic lesion severity was quantified using the Gensini score, which assigns weighted scores according to the degree of stenosis degree and the location of coronary segment location; higher scores indicate more extensive or severe coronary atherosclerosis. Gensini score was regarded as an angiographic severity indicator and comparator, but it was not included as a predictor in the primary pre-angiographic nomogram to avoid information leakage (13). Stable CAD was defined according to the 2024 ESC Guidelines for the Management of Chronic Coronary Syndromes (14). ACS subtypes, including UA, NSTEMI, and STEMI, were classified according to the 2023 ESC Guidelines for the Management of Acute Coronary Syndromes and the Fourth Universal Definition of Myocardial Infarction (15, 16), based on clinical presentation, electrocardiographic findings, cardiac troponin dynamics, and coronary angiographic results. These clinical categories were used for exploratory subgroup analyses. We also recorded the specific type of ACS (STEMI, NSTEMI, or UA) for subgroup analysis. The control group included patients with symptoms suggestive of CAD or high cardiovascular risk but without notable coronary stenosis, including patients with coronary spasm but no obstructive CAD.

### 2.4 Inflammatory indices

Venous blood samples were collected on admission before intervention. Routine complete blood count was analyzed using an automated hematology analyzer. The SIRI was calculated as:

$$\mathrm{SIRI}=\frac{\mathrm{Neutrophil}\;\mathrm{count}\times \mathrm{Monocyte}\;}{\mathrm{Lymphocyte}\;\mathrm{count}}$$

To compare the discriminatory performance of SIRI with other inflammation-based indices, NLR, PLR, MLR, SII, and AISI were also calculated from admission blood cell counts.

The NLR was calculated as:

$$\mathrm{NLR}=\frac{\mathrm{Neutrophil}\;\mathrm{count}\;}{\mathrm{Lymphocyte}\;\mathrm{count}}$$

The PLR was calculated as:

$$\mathrm{PLR}=\frac{\mathrm{Platelet}\;\mathrm{count}\;}{\mathrm{Lymphocyte}\;\mathrm{count}}$$

The MLR was calculated as:

$$\mathrm{MLR}=\frac{\mathrm{Monocyte}\;\mathrm{count}\;}{\mathrm{Lymphocyte}\;\mathrm{count}}$$

The SII was calculated as:

$$\mathrm{SII}=\frac{\mathrm{Platelet}\;\mathrm{count}\;\times \;\mathrm{Neutrophil}\;\mathrm{count}}{\mathrm{Lymphocyte}\;\mathrm{count}}$$

The AISI was calculated as:

$$\mathrm{AISI}=\frac{\mathrm{Platelet}\;\mathrm{count}\times \mathrm{Neutrophil}\;\mathrm{count}\times \mathrm{Monocyte}\;\mathrm{count}\;}{\mathrm{Lymphocyte}\;\mathrm{count}}$$

All cell counts were absolute values (×10^9^/L). This index was recorded for each patient as our priority biomarker of interest.

### 2.5 Statistical analysis

Continuous variables were expressed as mean ± standard deviation or median with interquartile range (IQR) according to the distribution of the variable. Categorical variables were expressed as counts and percentages. Between-group comparisons for continuous variables were performed using *t*-tests, Mann–Whitney *U*-tests, Kruskal–Wallis tests with Dunn *post-hoc* comparisons where appropriate; categorical variables were compared using chi-square tests. For categorical risk-factor subgroup plots, SIRI was displayed as log_10_ (SIRI) to reduce the visual influence of high-value outliers, while rank-based statistical comparisons were performed using the original distribution.

Correlations between SIRI and clinical, biochemical, or angiographic parameters were assessed using Spearman correlation analysis. To address possible confounding, sensitivity analyses for key associations were further evaluated using regression models adjusted for clinically relevant variables, including age, sex, hypertension, diabetes, hyperlipidemia, body mass index (BMI), and low-density lipoprotein-cholesterol (LDL-C), where applicable. Subgroup analyses across CAD phenotypes were interpreted as exploratory because of unequal subgroup sample sizes.

Univariate and multivariable binary logistic regression analyses were used to identify independent predictors of angiographic CAD. Candidate variables were selected based on univariable *p* < 0.10 and clinical relevance, and the final parsimonious model retained variables that remained independently associated with CAD while representing distinct clinical domains. Multicollinearity among key predictors and comparators, including albumin, LVEF, SIRI, log_10_ cardiac troponin I (cTnI), and Gensini score, was assessed using the variance inflation factor (VIF), with VIF > 5 considered suggestive of considerable collinearity.

Based on the multivariate results, a final logistic regression model was constructed to predict the probability of CAD. Variables with *p* < 0.05 in the multivariable model were selected for construction of the final predictive model. The fitted logistic regression model was expressed as:

$$\mathrm{logit}(p)=\beta_{0}\;+\;\beta_{1}\cdot \mathrm{Albumin}+\beta_{2}\cdot \mathrm{LVEF}+\beta_{3}\cdot \mathrm{SIRI}$$

The *β* coefficients were estimated using the maximum likelihood estimation (MLE) method, which identifies the set of parameters that maximizes the likelihood of observing the given data. Each *β* coefficient represents the change in the log-odds of the outcome per unit increase in the corresponding predictor. Odds ratios (ORs) were derived by exponentiating the *β* values (*OR* *=* *e^β^*) and are presented with 95% confidence intervals (CIs). The final CAD prediction model was visualized using a nomogram.

Model discrimination was evaluated using receiver operating characteristic (ROC) curves, area under the curves (AUCs) with 95% confidence intervals (CIs), Youden index-derived cutoff values, sensitivity, and specificity. Pairwise AUC comparisons were performed using the DeLong test. The statistical significance of inflammatory-index AUCs was tested against the null value of 0.5, and related multiple comparisons were interpreted with attention to false discovery rate adjustment where applicable. ROC curves in the supplementary inflammatory-index analysis were smoothed for visualization only, whereas AUCs, CIs, thresholds, sensitivity, and specificity were derived from the original empirical ROC curves.

Internal validation was performed using 1,000 bootstrap resamples, and calibration was assessed using bootstrap-corrected calibration curves, calibration intercept, calibration slope, and Brier score. Decision curve analysis was used to evaluate net benefit across threshold probabilities from 0.05 to 0.85. The added predictive value of SIRI was assessed by comparing the albumin + LVEF base model with the albumin + LVEF + SIRI full model using delta AUC, DeLong test, likelihood-ratio test, AIC (Akaike Information Criterion), BIC (Bayesian Information Criterion), integrated discrimination improvement, continuous net reclassification improvement, and decision curve analysis.

Model stability was evaluated by replacing untransformed SIRI values with log_10_ SIRI or SIRI quartiles. Subgroup ROC and decision curve analyses compared stable CAD, UA, NSTEMI, STEMI, and overall CAD with the non-CAD control group.

Analyses were performed using SPSS version 26.0 (IBM Corp., Armonk, NY, USA) and R version 4.5.2 (R Foundation for Statistical Computing, Vienna, Austria), and two-sided *p* values less than <0.05 was were considered statistically significant unless otherwise specified. Figures were drawn with GraphPad Prism 8.0 (GraphPad Software, San Diego, CA, USA).

## 3 Results

### 3.1 Baseline characteristics

Baseline characteristics of the study population are presented in Table 1, and the patient screening process is shown in Figure 1. A total of 394 patients were included in the final analysis. The mean age was 62.8 ± 12.0 years, 72.3% were male, and the mean BMI was 24.7 ± 3.4 kg/m^2^. Hypertension, diabetes mellitus, and hyperlipidemia were present in 61.4%, 26.6%, and 23.1% of patients, respectively. The clinical categories were control, stable CAD, UA, NSTEMI, and STEMI. The median (IQR) Gensini score, LVEF, and SIRI were 20.0 (5.0–47.3), 65% (62%–68%), and 1.1 (0.7–1.8), respectively.

**Table 1:** Baseline characteristics of participants.

Variable	Total (*N* = 394)	Variable	Total (*N* = 394)
Age (years)	62.8 ± 12.0	WBC (×10^9^/L)	6.5 (5.3–8.2)
Gender, *n* (%)		NE%	64.4 ± 10.2
Male	285 (72.3%)	LY%	26.2 ± 8.8
Female	109 (27.7%)	MO%	7.0 (6.0–8.0)
Diabetes, *n* (%)	105 (26.6%)	NE (×10^9^/L)	4.1 (3.1–5.5)
Hypertension, *n* (%)	242 (61.4%)	LY (×10^9^/L)	1.6 (1.3–2.1)
Hyperlipemia, *n* (%)	91 (23.1%)	MO (×10^9^/L)	0.4 (0.3–0.6)
Weight (kg)	65.1 (59.0–74.0)	PLT (×10^9^/L)	212.0 (179.8–243.3)
Height (cm)	165.0 (158.5–170.0)	cTnI (ng/mL)	0.0 (0.0–0.4)
BMI (kg/m^2^)	24.7 ± 3.4	BNP (pg/mL)	119.5 (46.0–468.0)
Systolic pressure (mmHg)	136.0 (125.0–149.0)	INR	1.0 (0.9–1.0)
Diastolic pressure (mmHg)	82.2 ± 11.7	APTT (s)	35.8 (33.7–38.2)
LVEF (%)	65 (62–68)	FIB (g/L)	3.2 (2.7–3.7)
		DD (mg/L)	0.2 (0.2–0.4)
CAD group, *n* (%)		ALT (U/L)	23.0 (16.0–34.0)
Control	94 (23.9%)	AST (U/L)	24.0 (19.0–33.0)
Non-MI CHD	175 (44.4%)	ALP (U/L)	86.0 (70.0–108.0)
MI CHD	125 (31.7%)	GGT (U/L)	30.0 (19.0–54.0)
Group (original), *n* (%)		Albumin (g/L)	41.8 (39.2–44.0)
Control	94 (23.9%)	Total bilirubin (*μ*mol/L)	11.7 (9.0–15.9)
UA	83 (21.1%)	Direct bilirubin (μmol/L)	2.4 (1.8–3.5)
S-CAD	88 (22.3%)	Indirect bilirubin (μmol/L)	9.3 (7.5–12.5)
NSTEMI	42 (10.7%)	Glu (mmol/L)	6.57 (5.30–8.66)
STEMI	87 (22.0%)	HBA1C (%)	5.9 (5.5–6.8)
CHD grade, *n* (%)		Cr (μmol/L)	70.2 (59.0–82.1)
Coronary sclerosis	94 (23.9%)	Creatine kinase (U/L)	109.0 (74.0–184.3)
Mild CHD	51 (12.9%)	LDH (U/L)	178.0 (153.8–213.0)
Severe CHD	249 (63.2%)	Triglyceride (mmol/L)	1.8 (1.2–2.7)
Coronary lesion, *n* (%)		Total cholesterol (mmol/L)	4.8 (3.9–5.7)
None	94 (23.9%)	HDL (mmol/L)	1.1 (0.9–1.3)
Single vessel disease	105 (26.6%)	LDL-c (mmol/L)	3.0 (2.5–3.6)
Multiple vessel disease	195 (49.5%)	apoA (g/L)	1.2 (1.1–1.3)
		apoB (g/L)	0.9 (0.8–1.2)
Gensini score	20.0 (5.0–47.3)	Cholinesterase (U/L)	8,818.5 (6,962.3–9,481.8)
SIRI	1.1 (0.7–1.8)	Kalium (mmol/L)	3.9 (3.7–4.1)

Baseline demographic, clinical, laboratory, echocardiographic, and angiographic characteristics of the study population. The table summarizes age, sex, body mass index, cardiovascular risk factors, routine laboratory findings, inflammatory markers, cardiac injury or stress markers, LVEF, Gensini score, and CAD phenotype distribution. S-CAD, stable coronary artery disease; UA, unstable angina; NSTEMI, non-ST-segment elevation myocardial infarction; STEMI, ST-segment elevation myocardial infarction; CAD, coronary artery disease; CHD, coronary heart disease; MI, myocardial infarction; SIRI, systemic inflammatory response index.

There were significant differences in age, sex, hyperlipidemia, inflammatory markers, myocardial injury markers, and Gensini score across the five clinical groups. SIRI showed a stepwise pattern across CAD phenotypes, with lower median values in controls and angina presentations and higher values in myocardial infarction groups. Median SIRI values were 0.78 in controls, 0.96 in stable CAD, 0.87 in UA, 1.59 in NSTEMI, and 2.62 in STEMI (*p* < 0.001).

### 3.2 Comparison of clinical features and indicators of patients with different CAD diagnosis

Supplementary Table S1 compares clinical and laboratory parameters among the five subgroups: Non-CAD, S-CAD, UA, NSTEMI, and STEMI. Gender and hypertension differed significantly among groups.

A significant difference in distribution of gender was observed among the five groups [male: Control 52 (55.3%), S-CAD 63 (71.6%), UA 60 (69.0%), NSTEMI 73 (88.0%), STEMI 37 (88.1%); *χ*^2^ = 29.447, *p* < 0.001]. In contrast, the distribution of hypertension (*p* = 0.082) and diabetes (*p* = 0.567) did not differ significantly among groups. However, a significant difference was observed in the prevalence of hyperlipemia [Control 44 (46.8%), S-CAD 20 (22.7%), UA 16 (18.4%), NSTEMI 9 (10.8%), STEMI 2 (4.8%); *χ*^2^ = 45.812, *p* < 0.001].

SIRI increased progressively across CAD phenotypes, with the lowest values observed in the control group [0.78 (0.60–1.13)] and UA group [0.87 (0.68–1.34)], intermediate values in the S-CAD group [0.96 (0.64–1.49)], higher values in the NSTEMI group [1.59 (1.06–2.37)], and the highest values in the STEMI group [2.62 (1.57–3.62)] (*p* < 0.001). These analyses suggest that the elevated SIRI, a marker of heightened systemic inflammation, was associated with a higher probability of myocardial infarction rather than angina.

Age differed significantly across groups (Control 62.23 ± 12.45; S-CAD 66.44 ± 9.79; UA 64.85 ± 10.34; NSTEMI 60.64 ± 13.59; STEMI 56.36 ± 12.05; *p* < 0.001). Compared with control, MI patients had lower HDL-C levels [Control 1.21 (1.04, 1.36); NSTEMI 1.02 (0.83, 1.19); STEMI 1.03 (0.89, 1.23); *p* < 0.001], and higher LDL-C, creatine kinase, LDH, and Gensini score compared with control and angina groups (all *p* < 0.05). Additionally, inflammatory markers including white blood cells count, neutrophil percent, neutrophil count, and monocyte count were significantly elevated in MI groups (all *p* < 0.001). Clinically, this may indicate that a heightened inflammatory response contributes to plaque instability, thrombosis formation, and progression of coronary artery disease.

### 3.3 Correlation and subgroup analyses of SIRI

Spearman analysis showed that SIRI was positively correlated with Gensini score in patients with CAD (*R*^2^ = 0.04, *p* < 0.001) but not in controls (*R*^2^ < 0.01, *p* = 0.997) (Supplementary Figure S1, Table 2). We also investigated the correlation between the SIRI and various clinical and biochemical parameters. Among metabolic indicators, triglyceride showed a significant positive correlation with SIRI in CAD patients (*R*^2^ = 0.02, *p* = 0.028), but no significant association was found in controls (*R*^2^ = 0.01, *p* = 0.274). In the CAD group, inflammatory and tissue injury markers demonstrated stronger associations: cTnI exhibited the strongest correlation with SIRI (*R*^2^ = 0.19, *p* < 0.001), followed by creatine kinase (*R*^2^ = 0.12, *p* < 0.001) and b-type natriuretic peptide (BNP) (*R*^2^ = 0.10, *p* < 0.001). Liver-derived proteins displayed inverse associations with SIRI. Both of Apolipoprotein A (apoA) (*R*^2^ = 0.03, *p* = 0.004) and albumin (*R*^2^ = 0.02, *p* = 0.007) showed a significant negative correlation in CAD groups, suggesting a potential inverse relationship between hepatic synthetic function and systemic inflammation. For coagulation-related markers, fibrinogen was positively correlated with SIRI in both CAD (*R*^2^ = 0.03, *p* = 0.003) and control groups (*R*^2^ = 0.04, *p* = 0.045). In CAD patients, APTT (*R*^2^ = 0.08, *p* < 0.001) and INR (*R*^2^ = 0.04, *p* < 0.001) were positively correlated with SIRI. Cardiac function parameters showed mild associations. LVEF demonstrated a weak inverse correlation with SIRI in CAD patients (*R*^2^ = 0.04, *p* < 0.001).

**Table 2:** Correlations of SIRI with clinical and biochemical parameters.

Variable	Control Group		Clinical CAD Group	
	*R* ^2^	*p*-value	*R* ^2^	*p*-value
Weight (kg)	0.01	0.320	0.01	0.059
Height (cm)	<0.01	0.670	0.04	<0.001^#^
BMI (kg/m^2^)	<0.01	0.608	<0.01	0.462
Systolic pressure (mmHg)	<0.01	0.825	0.01	0.073
Diastolic pressure (mmHg)	<0.01	0.478	<0.01	0.417
LVEF (%)	<0.01	0.351	0.04	<0.001^#^
WBC (*10^9^/L)	0.27	<0.001*	0.42	<0.001^#^
NE (%)	0.37	<0.001*	0.59	<0.001^#^
LY (%)	0.51	<0.001*	0.72	<0.001^#^
MO (%)	0.06	0.016*	<0.01	0.162
NE (*10^9^/L)	0.55	<0.001*	0.61	<0.001^#^
LY (*10^9^/L)	0.02	0.192	0.11	<0.001^#^
MO (*10^9^/L)	0.36	<0.001*	0.36	<0.001^#^
PLT (*10^9^/L)	0.01	0.342	0.02	0.008^#^
cTnI (ng/mL)	0.02	0.199	0.19	<0.001^#^
BNP (pg/mL)	<0.01	0.718	0.10	<0.001^#^
INR	<0.01	0.639	0.04	<0.001^#^
APTT (s)	<0.01	0.401	0.08	<0.001^#^
FIB (g/L)	0.04	0.045*	0.03	0.003^#^
DD (μg/mL)	0.09	0.005*	0.01	0.036^#^
ALT (U/L)	<0.01	0.648	<0.01	0.454
AST (U/L)	<0.01	0.898	0.05	<0.001^#^
ALP (U/L)	0.07	0.013*	<0.01	0.172
GGT (U/L)	<0.01	0.903	<0.01	0.336
Albumin (g/L)	<0.01	0.397	0.02	0.007^#^
Totalbilirubin (μmol/L)	<0.01	0.357	0.01	0.071
Directbilirubin (μmol/L)	<0.01	0.482	<0.01	0.134
Indirectbilirubin (μmol/L)	<0.01	0.424	<0.01	0.093
Glu (mmol/L)	0.12	<0.001*	<0.01	0.529
Cr (μmol/L)	0.02	0.223	<0.01	0.628
Creatine kinase (U/L)	<0.01	0.350	0.12	<0.001^#^
LDH (U/L)	0.01	0.286	0.07	<0.001^#^
Triglyceride (mmol/L)	0.01	0.274	0.02	0.028^#^
Total cholesterol (mmol/L)	<0.01	0.645	<0.01	0.422
HDL (mmol/L)	<0.01	0.415	<0.01	0.215
LDLc (mmol/L)	<0.01	0.892	0.01	0.069
apoA (g/L)	0.02	0.207	0.03	0.004^#^
apoB (g/L)	<0.01	0.828	0.01	0.067
Cholinesterase (U/L)	<0.01	0.845	0.01	0.056
Kalium (mmol/L)	<0.01	0.643	<0.01	0.102
HBA1C (%)	0.13	<0.001*	<0.01	0.498
Gensini	<0.01	0.997	0.04	<0.001^#^

Comparative analysis of the correlation between systemic inflammatory response index (SIRI) and clinical/biochemical parameters among CAD and control groups.

* *p* < 0.05, significant correlation in the control group.

# *p* < 0.05, significant correlation in the CAD group.

Supplementary Figure S2 uses log_10_ SIRI on the *y*-axis to improve the visualization of SIRI distributions across categorical risk factors. Mann–Whitney *U*-test was used to compare SIRI levels stratified by key cardiometabolic risk factors. SIRI was significantly higher in CAD patients than controls in both males (*p* = 0.001) and females (*p* = 0.001), indicating that the association between systemic inflammatory burden and CAD was not sex-specific. No significant differences in SIRI were observed according to diabetes status (control, *p* = 0.239; CAD, *p* = 0.300) or hypertension status (control, *p* = 0.078; CAD, *p* = 0.631). For hyperlipidemia, SIRI did not differ significantly in the control group (*p* = 0.742), but a significant difference was observed in the CAD group (*p* = 0.013).

### 3.4 Comparison of SIRI with other inflammation-based indices

To strengthen the rationale for focusing on SIRI, we compared its CAD-discriminatory performance with NLR, PLR, MLR, SII, and AISI. SIRI showed the highest numerical AUC among the six indices (AUC = 0.684, 95% CI: 0.625–0.744, *p* < 0.001), followed by NLR (AUC = 0.679), AISI (AUC = 0.657), SII (AUC = 0.647), MLR (AUC = 0.635), and PLR (AUC = 0.543). The optimal SIRI cut-off by based on the Youden index was 1.205, with a sensitivity of 48.7% and specificity of 81.9% (Supplementary Table S2 and Figure S3).

Pairwise DeLong comparisons showed that the AUC of SIRI was significantly higher than that of PLR (*p* < 0.001) and MLR (*p* = 0.010), whereas no significant difference was observed between SIRI and NLR (*p* = 0.791), SII (*p* = 0.152), or AISI (*p* = 0.069) (Supplementary Table S3). These findings support the selection of SIRI as the principal inflammatory index in this cohort while avoiding the overstatement that SIRI is statistically superior to all related inflammatory indices.

### 3.5 Regression model and nomogram development

Univariate logistic regression (Table 3) identified variables associated with CAD presence, including LVEF (OR ≈ 0.000, 95% CI: 0.000–0.001, *p* = 0.022), BNP (OR = 0.877, 95% CI: 0.000–0.998, *p* = 0.002), apoA (OR = 1.157, 95% CI: 0.991–1.352, *p* = 0.065), platelet count (OR = 0.957, 95% CI: 0.922–0.994, *p* = 0.023), serum albumin (OR = 0.895, 95% CI: 0.842–0.952, *p* = 0.011), and SIRI (OR = 1.121, 95% CI: 1.056–2.874, *p* = 0.009).

**Table 3:** Univariate and multivariate logistic regression analyses for CAD.

Variable	Univariate logistic regression analyses		Multivariate logistic regression analyses	
	OR (95% CI)	*p*-value	OR (95% CI)	*p*-value
LVEF (%)	0.000 (0.000–0.001)	0.022	0.000 (0.000–0.056)	0.003*
cTnI (ng/mL)	1.837 (0.712–4.741)	0.209	—	—
BNP (pg/mL)	0.877 (0.000–0.998)	0.002	0.761 (0.370–1.560)	0.459
INR	1.043 (0.243–1.388)	0.662	—	—
APTT (s)	1.041 (0.632–1.713)	0.875	—	—
AST (U/L)	1.008 (0.842–1.206)	0.934	—	—
Creatinekinase (U/L)	0.996 (0.980–1.012)	0.641	—	—
LDH (U/L)	1.008 (0.967–1.050)	0.701	—	—
apoA (g/L)	1.157 (0.991–1.352)	0.065	0.961 (0.770–1.566)	0.458
PLT (×10^9^/L)	0.957 (0.922–0.994)	0.023	1.000 (0.995–1.004)	0.899
FIB (g/L)	4.931 (0.720–33.784)	0.104	—	—
Albumin (g/L)	0.895 (0.842–0.952)	0.011	0.916 (0.853–0.985)	0.017*
Triglyceride (mmol/L)	0.514 (0.090–2.946)	0.455	—	—
SIRI	1.121 (1.056–2.874)	0.009	1.828 (1.194–2.798)	0.006*

Univariate and multivariable logistic regression analyses for angiographic CAD. Variables with *p* < 0.10 in the univariate analysis were included in the multivariate analysis, and those with *p* < 0.05 in the multivariate model were selected to construct the final logistic regression model.

*Statistically significant at the 0.05 level (*p* < 0.05).

Multivariate logistic regression confirmed that albumin, LVEF, and SIRI were independently associated with CAD presence. These three variables were incorporated into the final predictive models for both CAD risk.

The fitted logistic regression equation for CAD probability was:

logit(p)=8.985−0.087×albumin−8.248×LVEF+0.603×SIRI

**Figure 2:**
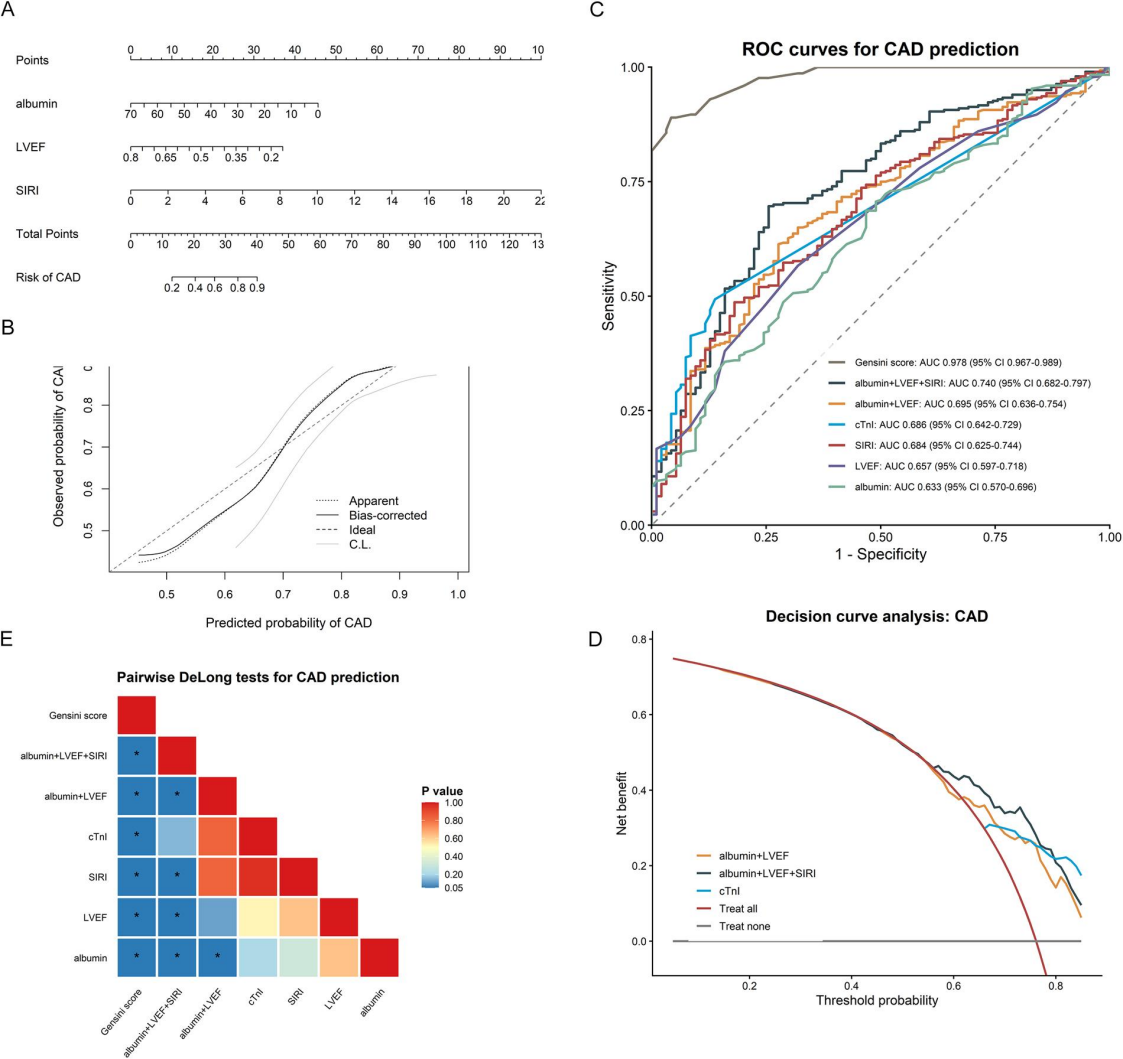
Nomogram and internal performance assessment of the albumin + LVEF + SIRI model for angiographic CAD prediction. **(A)** Nomogram constructed from multivariable logistic regression using albumin, LVEF, and SIRI to estimate the individual probability of angiographic CAD. Each predictor corresponds to a point score, and the total score is mapped to the predicted probability of CAD. **(B)** ROC curves comparing the albumin + LVEF + SIRI model with the albumin + LVEF base model and individual indicators, including albumin, LVEF, SIRI, cTnI, and Gensini score. Gensini score was included only as an angiographic reference comparator and was not used in the primary preangiographic nomogram. **(C)** Bootstrap-corrected calibration curve based on 1,000 resamples, showing agreement between predicted and observed probability of CAD. The ideal line represents perfect calibration. **(D)** Decision curve analysis comparing the net benefit of the albumin + LVEF + SIRI model, albumin + LVEF model, cTnI, treat-all strategy, and treat-none strategy across threshold probabilities from 0.05 to 0.85. **(E)** Pairwise DeLong test heatmap comparing AUC differences among Gensini score, the albumin + LVEF + SIRI model, the albumin + LVEF model, cTnI, SIRI, LVEF, and albumin. The color scale represents *p* values for pairwise comparisons, and asterisks indicate statistically significant differences. CAD, coronary artery disease; LVEF, left ventricular ejection fraction; SIRI, systemic inflammatory response index; cTnI, cardiac troponin I; ROC, receiver operating characteristic.

The prediction analysis focused on angiographic CAD as the only primary outcome. Gensini score was used as a severity indicator and comparator rather than as an outcome for nomogram construction. A nomogram based on albumin, LVEF, and SIRI was retained to provide an individualized visual representation of the preliminary CAD risk model (Figure 2A). The calibration curve showed acceptable agreement between predicted and observed CAD probability (Figure 2B).

### 3.6 Internal validation, model comparison, and clinical Net benefit

The incremental value of SIRI was further evaluated by comparing the albumin + LVEF base model with the full albumin + LVEF + SIRI model (Table 4). The final albumin + LVEF + SIRI model showed moderate discrimination for angiographic CAD, with an AUC of 0.740 (95% CI: 0.682–0.797), sensitivity of 69.7%, and specificity of 74.5%. This model outperformed the albumin + LVEF base model, which had an AUC of 0.695 (95% CI: 0.636–0.754). Addition of SIRI increased the AUC by 0.045 (DeLong *p* = 0.024), improved model fit by likelihood-ratio testing (*p* < 0.001), reduced AIC from 410.21 to 394.76 and BIC from 422.14 to 410.66, and significantly improved likelihood-based performance (likelihood-ratio test *p* < 0.001).

**Table 4:** Added predictive value of SIRI for angiographic CAD prediction.

Base model	Full model	Base AUC	Full AUC	ΔAUC	DeLong P	ΔAIC	ΔBIC	LRT P	Mean ΔNB	Median ΔNB	Max ΔNB
Albumin + LVEF	albumin + LVEF + SIRI	0.695	0.740	+0.045	0.024	−15.45	−11.47	<0.001	0.017	0.003	0.079

ROC analysis for overall CAD prediction. Δ values were calculated as full model minus base model; negative ΔAIC and ΔBIC indicate improved model fit. ΔNB was derived from decision curve analysis across threshold probabilities of 0.05–0.85. LVEF, left ventricular ejection fraction; AIC, Akaike information criterion; BIC, Bayesian information criterion; CAD, coronary artery disease; LRT, likelihood-ratio test; NB, net benefit; SIRI, systemic inflammatory response index.

ROC analysis for overall angiographic CAD prediction is presented in Table 5 and Figure 2C. Gensini score showed the highest AUC (0.978, 95% CI: 0.967–0.989), as expected because it is derived from angiographic findings; therefore, it was interpreted only as an angiographic reference comparator. Among pre-angiographic predictors, the albumin + LVEF + SIRI model achieved the highest AUC (0.740, 95% CI: 0.682–0.797, *p* < 0.001), with a sensitivity of 69.7% and specificity of 74.5% at the optimal threshold. This performance was higher than that of the albumin + LVEF base model (AUC = 0.695, 95% CI: 0.636–0.754), SIRI alone (AUC = 0.684, 95% CI: 0.625–0.744), cTnI (AUC = 0.686, 95% CI: 0.642–0.729), LVEF alone (AUC = 0.657, 95% CI: 0.597–0.718), and albumin alone (AUC = 0.633, 95% CI: 0.570–0.696). Decision curve analysis also showed a positive net-benefit gain after adding SIRI (mean ΔNB = 0.017; median ΔNB = 0.003; maximum ΔNB = 0.079) across threshold probabilities of 0.05–0.85 (Figure 2D). Pairwise DeLong comparisons among the main ROC curves are shown in Figure 2E.

**Table 5:** ROC analysis for overall angiographic CAD prediction.

Predictor/model	AUC (95% CI)	*p* value	Optimal threshold	Sensitivity (%)	Specificity (%)
Gensini score	0.978 (0.967–0.989)	<0.001	7.75	89.0	95.7
Albumin + LVEF + SIRI	0.740 (0.682–0.797)	<0.001	0.731	69.7	74.5
Albumin + LVEF	0.695 (0.636–0.754)	<0.001	0.76	61.3	72.3
cTnI	0.686 (0.642–0.729)	<0.001	−1.818	49.3	86.2
SIRI	0.684 (0.625–0.744)	<0.001	1.205	48.7	81.9
LVEF	0.657 (0.597–0.718)	<0.001	−0.655	56.7	68.1
Albumin	0.633 (0.570–0.696)	<0.001	−43.05	68.7	53.2

ROC analysis for overall angiographic CAD prediction. The optimal threshold, sensitivity, and specificity were determined using the Youden index. Gensini score was included only as an angiographic reference comparator and was not used in the primary pre-angiographic nomogram. For LVEF and albumin, inverse coding was used in ROC analysis so that higher transformed values indicated higher CAD probability; cTnI was analyzed after log10 transformation. AUC, area under the receiver operating characteristic curve; CAD, coronary artery disease; CI, confidence interval; cTnI, cardiac troponin I; LVEF, left ventricular ejection fraction; ROC, receiver operating characteristic; SIRI, systemic inflammatory response index.

### 3.7 Stability of CAD prediction models and subgroup analyses across CAD phenotypes

Model stability analysis showed consistent discrimination when SIRI was modeled as using a the untransformed raw continuous variable, a log10-transformed variable, or a quartile-based variable. (Supplementary Tables S4,S5 and Figure S4). The AUCs of the corresponding multivariable models were 0.740, 0.740, and 0.742, respectively. Univariatble ROC curves for untransformed raw SIRI values, log_10_ (SIRI), and SIRI quartiles were also comparable, with AUCs of 0.684, 0.684, and 0.680, respectively. These results indicate that the association between SIRI and CAD prediction was robust to different scaling strategies; raw SIRI was therefore retained in the main model for interpretability.

**Figure 3:**
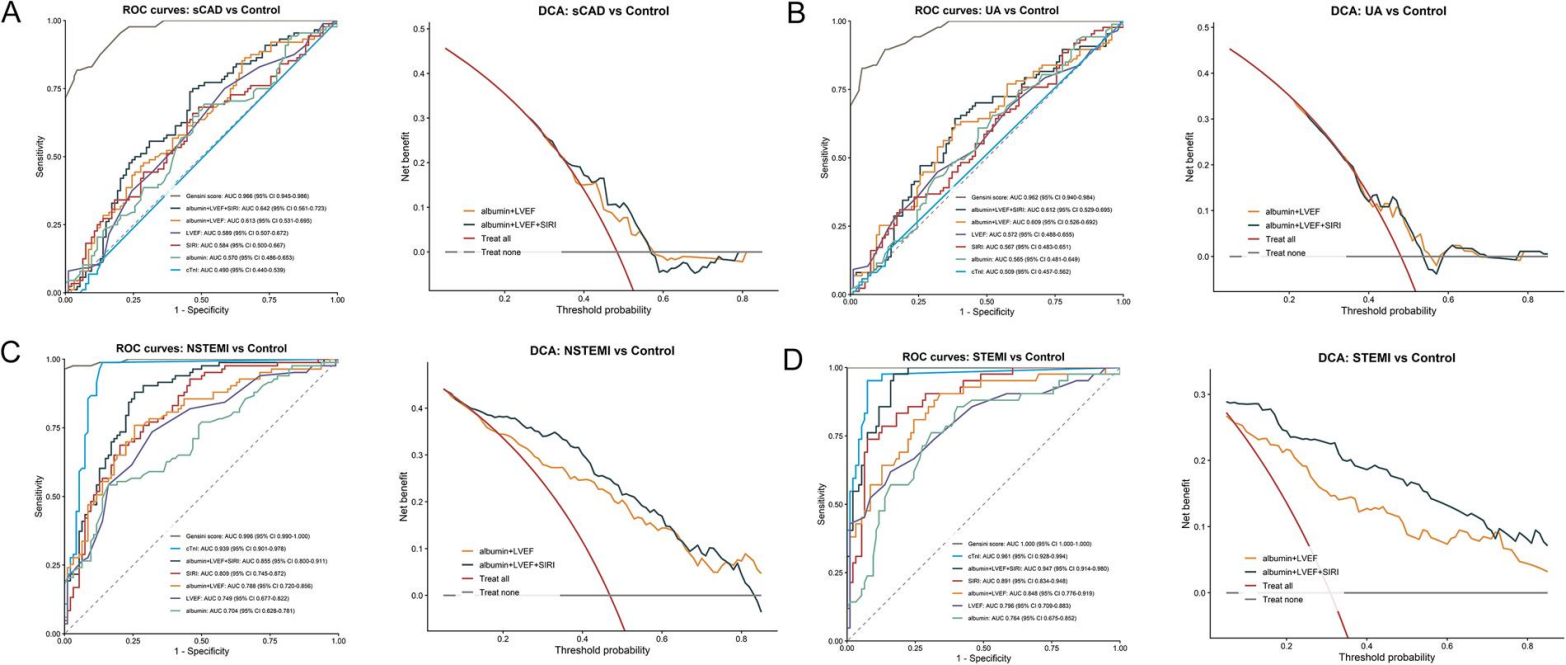
Subgroup ROC and decision curve analyses across CAD phenotypes. ROC curves and decision curve analyses were performed by comparing each CAD phenotype with the non-CAD control group. **(A)** Stable CAD vs. control. **(B)** Unstable angina vs. control. **(C)** NSTEMI vs. control. **(D)** STEMI vs. control. The albumin + LVEF + SIRI model was compared with albumin + LVEF, albumin, LVEF, SIRI, cTnI, and Gensini score. DCA was performed across threshold probabilities from 0.05 to 0.85. S-CAD, stable coronary artery disease; UA, unstable angina; NSTEMI, non ST-segment elevation myocardial infarction; STEMI, ST-segment elevation myocardial infarction.

Subgroup ROC analyses comparing each CAD phenotype with the non-CAD control group are shown in Supplementary Table S6 and Figure 3. The albumin + LVEF + SIRI model showed modest discrimination for stable CAD (AUC = 0.642) and UA (AUC = 0.612), with AUCs of 0.642 and 0.612, respectively, but stronger discrimination for NSTEMI (AUC = 0.855) and STEMI (AUC = 0.947), with AUCs of 0.855 and 0.947. In contrast, cTnI showed limited discrimination in stable CAD and UA but strong discrimination in NSTEMI and STEMI, consistent with its role as a myocardial injury marker rather than a general CAD screening marker. These results suggest that model performance may differ across CAD phenotypes and that future validation should be phenotype-specific and the model is better suited for detecting more inflammatory or infarction CAD phenotypes.

## 4 Discussion

This study demonstrated that admission SIRI was independently associated with the presence of angiographic CAD, greater angiographic disease burden, and clinical presentations across the ACS spectrum in an angiography-based cohort from southeastern China. Among several inflammatory indices, SIRI showed the highest numerical discrimination for CAD, although it was not statistically superior to all closely related indices. Albumin, LVEF, and SIRI remained independently associated with CAD and formed a simple model with moderate discrimination, acceptable internal calibration, and stable performance across alternative SIRI scaling methods. These findings support SIRI as a biologically plausible and practical inflammatory marker, while its clinical value should be interpreted cautiously, and SIRI should be considered complementary to established clinical assessment rather than a substitute for conventional diagnostic strategies or coronary angiography.

Consistent with previous findings from Chinese cohorts, our single-center data confirmed that SIRI was independently correlated with CAD prevalence and angiographic severity. The nomogram combining SIRI, albumin and LVEF showed moderate predictive performance. This combined model may serve as a simple tool for CAD risk stratification in local hospitalized patients. Regional characteristics may explain the prominent predictive value of inflammatory markers in this cohort. The population-based Zhejiang survey using 24-hour urinary sodium documented substantial sodium exposure (mean salt intake ≈ 9.68 g/day) and a hypertension prevalence of ∼30% in adults, supporting the plausibility that high-salt dietary patterns contribute to a large hypertensive substrate in this province (17). Our cohort's clinical characteristics align with contemporary Chinese angiography cohorts investigating SIRI in CAD, where SIRI levels correlated with CAD prevalence and angiographic stenosis severity in Wuhan and Hubei cohorts (18, 19). Unlike Western cohorts, where metabolic syndrome components dominate cardiovascular risk stratification (20–23), our population exhibited distinct features: lower mean BMI, relatively lower LDL-C levels, but a higher prevalence of diabetes and reduced HDL-C. Traditional lipid parameters did not retain independent predictive value in multivariate analysis, whereas inflammatory and nutritional markers emerged as dominant predictors. Together, these comparisons suggest that while SIRI–CAD associations are reproducible across geography, their effect sizes and the relative importance of traditional risk factors depend on population age, comorbidity, and clinical presentation.

Atherosclerosis is a chronic inflammatory disease in which innate immune activation, maladaptive immune regulation, and inflammatory mediators promote plaque growth and destabilization. Contemporary reviews emphasize key roles for mononuclear phagocytes across all stages of atherosclerosis and highlight how innate and adaptive immune mechanisms can accelerate or curb lesion development (24, 25). SIRI therefore represents a biologically coherent proxy for innate effector activation relative to adaptive immune reserve. Neutrophils may promote oxidative stress, endothelial dysfunction, and plaque destabilization; monocytes participate in macrophage accumulation, foam cell formation, and inflammatory plaque remodeling; and lymphocyte reduction may reflect impaired immune regulation or stress-related immune imbalance. Previous literature has proven that SIRI has robust predictive efficacy for CAD and acute coronary events in both young and elderly populations (6, 7). However, PLR and SII cannot independently predict CAD in general populations after confounding adjustment (8). As an easy-to-obtain inflammatory marker that mirrors inflammation and immune regulation, SIRI exhibits more consistent clinical performance across different crowds, which is why we selected it as our core study indicator. Nevertheless, the overlap between SIRI and other blood cell-derived indices indicates that SIRI should not be considered a replacement for established assessment strategies or a universally superior inflammatory marker.

The observed associations between SIRI, Gensini score, cTnI, creatine kinase, BNP, and coagulation-related markers are consistent with a link between systemic inflammation, plaque burden, thrombo-inflammatory activation, and myocardial injury. Our correlation analyses (Table 2) support this mechanistic linkage and show patterns partly concordant with prior SIRI–CAD studies. In CAD patients, SIRI was positively correlated with angiographic severity (Gensini score), myocardial injury/stress markers, coagulation indices, and triglyceride, while negatively correlated with apoA. These findings are directionally consistent with external angiography cohorts, where higher SIRI was associated with greater coronary lesion burden, as reported in a Hubei cohort (19) and a study of young symptomatic CAD patients (26). However, not all correlates were identical across studies. Wang et al. reported a clear negative association between SIRI and HDL in young symptomatic CAD (26), whereas HDL showed a weak and non-significant relationship with SIRI in our CAD group. Similarly, the Wuhan angiography cohort reported the strongest diagnostic performance for the composite SIRI/HDL index (AUC = 0.680) and highlighted stronger correlations of SIRI/HDL with Gensini and SYNTAX scores than SIRI alone (18). Several factors may explain cross-study differences: variations in case mix (stable CAD, ACS, or mixed cohorts), medication-related confounding (e.g., statin exposure), comorbidity-dependent inflammatory baseline status, and differences in SIRI measurement and statistical scaling approaches. These considerations are critical for interpreting effect sizes and translating SIRI into clinical decision-making tools. In multivariable logistic regression (Table 3), albumin, LVEF, and SIRI remained independent predictors of CAD. The nomogram based on this three-variable triad showed favorable discrimination and calibration for CAD presence, as validated by bootstrap resampling (Figure 2).

Serum albumin is not only a key protein for maintaining plasma colloid osmotic pressure, but also one of the most important endogenous antioxidants and anti-inflammatory molecules. In the setting of CAD, hypoalbuminemia usually indicates malnutrition, consumption caused by chronic inflammation and markedly elevated oxidative stress (27, 28). Albumin exerts cardioprotective effects by scavenging free radicals, inhibiting platelet aggregation and binding toxic metabolites. A reduction in albumin makes vascular walls more vulnerable to oxidative damage, thereby facilitating the development of atherosclerotic plaques (28). Multiple studies have demonstrated that in elderly patients with stable CAD, hypoalbuminemia predicts a higher risk of subsequent ACS and acts as an independent marker for increased long-term cardiovascular mortality and poor prognosis (7, 27). Different from inflammatory and nutritional indicators, LVEF is a classic echocardiographic parameter that directly evaluates left ventricular systolic function. Impaired LVEF often results from chronic myocardial ischemia or prior myocardial injury, and it has long been recognized as a powerful prognostic marker for CAD patients. Studies have shown that decreased LVEF predicts elevated all-cause mortality and a higher risk of major adverse cardiovascular events (MACE) in CAD patients who receive percutaneous coronary intervention (PCI) (29).

The results of the subgroup analysis also highlight the need for careful interpretation. The model showed stronger discrimination in NSTEMI and STEMI than in stable CAD and UA, likely because acute myocardial infarction is accompanied by more pronounced inflammation, myocardial injury, and functional disturbance. In contrast, stable CAD and UA may be influenced by chronic plaque burden, endothelial dysfunction, vasospasm, medication exposure, and other factors that are less fully captured by the current three-variable model. The strong performance of cTnI in myocardial infarction subgroups further supports its role as an injury marker, whereas the albumin + LVEF + SIRI model may provide broader but less specific information across inflammatory, nutritional, and functional dimensions.

Compared with published SIRI-oriented diagnostic approaches, our model achieves comparable discrimination with fewer variables and adds functional and nutritional dimensions absent from many prior SIRI-only or SIRI/lipid-index models. A European angina-equivalent cohort reported AUC = 0.725 for SIRI at a cut-off of 1.22 (30). Notably, among patients with T2DM undergoing angiography, addition of SIRI to stress hyperglycemia ratio improved CAD discrimination (combined model AUC = 0.813) and was accompanied by bootstrapped calibration and improved reclassification (NRI/IDI) (31). Taken together, these data suggest that SIRI can contribute to CAD detection across populations, and that augmenting SIRI with context-specific clinical domains (injury markers, metabolic stress, or cardiac function) may enhance performance. Mechanistically, albumin is a negative acute-phase reactant reflecting systemic inflammation, nutritional reserve, and hepatic synthetic capacity; low albumin is associated with higher mortality in acute coronary syndromes in pooled analyses (32). LVEF reflects downstream myocardial consequence of ischemia/infarction and chronic remodeling, and it complements inflammatory indices by capturing organ-level impact. SIRI provides an immuno-inflammator*y* axis consistent with inflammation-driven plaque progression and complication biology (24). Regional characteristics may partly influence the relative contribution of inflammatory and host-reserve markers in this cohort. The Wenzhou inpatient population had a relatively modest BMI profile, and conventional lipid parameters did not dominate the final multivariable model. Dietary patterns, salt exposure, occupational factors, lifestyle habits, and environmental exposures may also affect systemic inflammation and CAD risk in this region. Zhejiang's high salt exposure and substantial hypertension prevalence (17) are compatible with a setting where endothelial stress and inflammatory activation are prominent, increasing the incremental value of inflammation and host-reserve markers. Additionally, the cohort's modest BMI (Table 1) suggests that overt obesity-driven dysmetabolism may be less dominant than in some Western settings, potentially shifting predictive weight toward inflammatory and functional domains in an inpatient angiography spectrum. These factors were not fully captured in the current retrospective dataset, but they provide an important rationale for future prospective multicenter studies that incorporate broader exposure variables and long-term outcomes.

### 4.1 Clinical implications

This study provides practical reference for daily clinical work in regional hospitals. SIRI is derived from routine blood tests, which is low-cost, easily accessible and widely available in primary and tertiary medical institutions. Combined with serum albumin and left ventricular ejection fraction, this simple three-indicator model can assist clinicians in preliminary risk stratification for patients undergoing coronary angiography. For patients with elevated SIRI, decreased albumin and reduced LVEF, physicians may pay closer attention to the possibility of coronary artery disease and severe coronary lesions, and prioritize further definitive examination and individualized intervention. Given the prominent role of inflammation in this southeastern Chinese cohort, the combined indicators are particularly suitable for auxiliary assessment of CAD risk among local inpatients.

### 4.2 Limitations

Several limitations should be acknowledged. First, this was a retrospective, single-center, angiography-referred study, which may introduce referral and selection bias and limit the generalizability of our findings to community-based or primary screening populations. Although model evaluation was strengthened using ROC comparison, bootstrap-corrected calibration, calibration metrics, decision curve analysis, stability testing, and subgroup analyses, these internal analyses cannot replace independent external validation. Moreover, the albumin + LVEF + SIRI model showed only moderate discrimination and should therefore be regarded as an exploratory risk-stratification tool rather than a definitive clinical model. We attempted to identify suitable public datasets for validation; however, available databases generally lacked the required combination of angiographic CAD classification, leukocyte subtypes for SIRI calculation, albumin, LVEF, myocardial injury markers, and angiographic severity measures. Future external validation in independent multicenter cohorts, including other regional hospitals and community-based populations, is essential before clinical application, and model reporting and validation should follow established guidance such as TRIPOD (33).

Secondly, residual confounding remains possible because detailed information on medication use, intercurrent infection, dietary patterns, salt intake, occupational or environmental exposure, lifestyle habits, and unmeasured inflammatory biomarkers such as hs-CRP and IL-6 was not systematically collected. In addition, this study was based on admission data and coronary angiography findings, and long-term post-angiography outcomes, including MACE, recurrent myocardial infarction, repeated revascularization, rehospitalization, all-cause mortality, and cardiovascular mortality, were unavailable. Therefore, further large-scale prospective multicenter studies with standardized follow-up, broader exposure assessment, and longitudinal clinical endpoints are warranted to validate, recalibrate, and refine this inflammation–albumin–cardiac function framework and to determine its prognostic value in CAD risk assessment.

## 5 Conclusion

SIRI was associated with CAD presence, angiographic severity, and myocardial injury markers. Albumin, LVEF, and SIRI were independently associated with angiographic CAD and formed a simple model with moderate discrimination, acceptable internal calibration, and stable performance across different SIRI scaling approaches. The triad-based nomogram exhibited moderate discrimination and good internal calibration for CAD prediction, as well as satisfactory performance for Gensini score evaluation. This work puts forward an exploratory hypothesis linking inflammation, nutrition and cardiac function, which requires further validation in larger cohorts.

## Data Availability

The data analyzed in this study is subject to the following licenses/restrictions: The clinical datasets utilized in this study are confidential and sourced from the hospital's electronic medical record system. All patient-related information was strictly de-identified and anonymized before analysis to protect individual privacy. Data sharing is restricted in accordance with relevant medical privacy laws and institutional data management regulations, and thus the original raw datasets cannot be publicly disclosed or shared. Requests to access these datasets should be directed to Lianpin Wu, 198019@wzhealth.com.

## References

[1] World Health Organisation. Global Health Estimates: Life expectancy and leading causes of death and disability (2021). Available online at: https://www.who.int/data/gho/data/themes/mortality-and-global-health-estimates (accessed April 14, 2026).

[2] Martin SS Aday AW Allen NB Almarzooq ZI Anderson CAM Arora P. 2025 Heart disease and stroke statistics: a report of US and global data from the American Heart Association. Circulation. (2025) 151(8):e41–e660. 10.1161/CIR.0000000000001303 39866113 PMC12256702

[3] Stojanovic SD Fiedler J Bauersachs J Thum T Sedding DG. Senescence-induced inflammation: an important player and key therapeutic target in atherosclerosis. Eur Heart J. (2020) 41(31):2983–96. 10.1093/eurheartj/ehz919 31898722 PMC7453834

[4] Liu Q Li Z Huang L Zhou D Fu J Duan H. Telomere and mitochondria mediated the association between dietary inflammatory index and mild cognitive impairment: a prospective cohort study. Immun Ageing. (2023) 20(1):1. 10.1186/s12979-022-00326-4 36604719 PMC9813461

[5] Tuzimek A Dziedzic EA Beck J Kochman W. Correlations between acute coronary syndrome and novel inflammatory markers (systemic immune-inflammation index, systemic inflammation response index, and aggregate index of systemic inflammation) in patients with and without diabetes or prediabetes. J Inflamm Res. (2024) 17:2623–32. 10.2147/JIR.S454117 38707954 PMC11067916

[6] Sun J Qi S Yu M Lei S Han W Wu S. The clinical significance and prognostic value of inflammatory hematological indices in young patients with coronary artery disease. J Inflamm Res. (2026) 19:1–19. 10.2147/JIR.S582913 PMC13274759 42318307

[7] Bao Q Liu T Song H Bao W Fan W. Prognostic role of inflammatory hematologic indices in predicting acute coronary syndrome in elderly patients with chronic coronary syndrome. J Inflamm Res. (2025) 18:9637–53. 10.2147/JIR.S528161 40727457 PMC12301150

[8] Sun T Chen P Zheng X. A retrospective analysis from NHANES 2003–2018 on the associations between inflammatory markers and coronary artery disease, all-cause mortality and cardiovascular mortality. PLoS One. (2025) 20(7):e0326953. 10.1371/journal.pone.0326953 40632706 PMC12240292

[9] Stergiou GS Palatini P Parati G O’Brien E Januszewicz A Lurbe E. 2021 European Society of Hypertension practice guidelines for office and out-of-office blood pressure measurement. J Hypertens. (2021) 39(7):1293–302. 10.1097/HJH.0000000000002843 33710173

[10] Cosentino F Grant PJ Aboyans V Bailey CJ Ceriello A Delgado V. 2019 ESC guidelines on diabetes, pre-diabetes, and cardiovascular diseases developed in collaboration with the EASD. Eur Heart J. (2020) 41(2):255–323. 10.1093/eurheartj/ehz486 31497854

[11] Mach F Baigent C Catapano AL Koskinas KC Casula M Badimon L. 2019 ESC/EAS guidelines for the management of dyslipidaemias: lipid modification to reduce cardiovascular risk. Eur Heart J. (2020) 41(1):111–88. 10.1093/eurheartj/ehz455 31504418

[12] Lawton JS Tamis-Holland JE Bangalore S Bates ER Beckie TM Bischoff JM. 2021 ACC/AHA/SCAI guideline for coronary artery revascularization: a report of the American College of Cardiology/American Heart Association Joint Committee on clinical practice guidelines. J Am Coll Cardiol. (2022) 79(2):e21–129. 10.1016/j.jacc.2021.09.006 34895950

[13] Rampidis GP Benetos G Benz DC Giannopoulos AA Buechel RR. A guide for Gensini score calculation. Atherosclerosis. (2019) 287:181–3. 10.1016/j.atherosclerosis.2019.05.012 31104809

[14] Vrints C Andreotti F Koskinas KC Rossello X Adamo M Ainslie J. 2024 ESC guidelines for the management of chronic coronary syndromes. Eur Heart J. (2024) 45(36):3415–537. 10.1093/eurheartj/ehae177 39210710

[15] Byrne RA Rossello X Coughlan JJ Barbato E Berry C Chieffo A. 2023 ESC guidelines for the management of acute coronary syndromes. Eur Heart J. (2023) 44(38):3720–826. 10.1093/eurheartj/ehad191 37622654

[16] Thygesen K Alpert JS Jaffe AS Chaitman BR Bax JJ Morrow DA. Fourth universal definition of myocardial infarction (2018). Rev Esp Cardiol. (2019) 72(1):72. 10.1016/j.jacc.2018.08.1038 30153967

[17] Du X Fang L Xu J Chen X Zhang J Bai Y. Prevalence, awareness, treatment and control of hypertension and sodium intake in Zhejiang Province, China: a cross-sectional survey in 2017. PLoS One. (2019) 14(12):e0226756. 10.1371/journal.pone.0226756 31869377 PMC6927602

[18] Peng A Zhang B Wang S Feng Y Liu S Liu C. Comparison of the value of various complex indexes of blood cell types and lipid levels in coronary heart disease. Front Cardiovasc Med. (2023) 10:1284491. 10.3389/fcvm.2023.1284491 38162141 PMC10754977

[19] He T Luo Y Wan J Hou L Su K Zhao J. Analysis of the correlation between the systemic inflammatory response index and the severity of coronary vasculopathy. Biomol Biomed. (2024) 24(6):1726–34. 10.17305/bb.2024.10747 38907736 PMC11496849

[20] D’Agostino RB Vasan S Pencina RS Wolf MJ Cobain PA Massaro M. General cardiovascular risk profile for use in primary care: the Framingham Heart Study. Circulation. (2008) 117(6):743–53. 10.1161/CIRCULATIONAHA.107.699579 18212285

[21] Menasché P Alfieri O Janssens S McKenna W Reichenspurner H Trinquart L. The myoblast autologous grafting in ischemic cardiomyopathy (MAGIC) trial: first randomized placebo-controlled study of myoblast transplantation. Circulation. (2008) 117(9):1189–200. 10.1161/CIRCULATIONAHA.107.734103 18285565

[22] Wilson PW D’Agostino RB Levy D Belanger AM Silbershatz H Kannel WB. Prediction of coronary heart disease using risk factor categories. Circulation. (1998) 97(18):1837–47. 10.1161/01.CIR.97.18.1837 9603539

[23] Wilson PW D’Agostino RB Parise H Sullivan L Meigs JB. Metabolic syndrome as a precursor of cardiovascular disease and type 2 diabetes mellitus. Circulation. (2005) 112(20):3066–72. 10.1161/CIRCULATIONAHA.105.539528 16275870

[24] Geovanini GR Libby P. Atherosclerosis and inflammation: overview and updates. Clin Sci. (2018) 132(12):1243–52. 10.1042/CS20180306 29930142

[25] Wolf D Ley K. Immunity and inflammation in atherosclerosis. Circ Res. (2019) 124(2):315–27. 10.1161/CIRCRESAHA.118.313591 30653442 PMC6342482

[26] Wang C Yan W Ren M Zhong L. Screening significance of systemic immune-inflammation index (SII) and systemic inflammation response index (SIRI) in coronary heart disease of symptomatic youth. Immun Inflamm Dis. (2024) 12(8):e1369. 10.1002/iid3.1369 39110067 PMC11304894

[27] Cheng CW Lee CW Chien SC Yeh HI Chen CY. Serum albumin was associated with a long term cardiovascular mortality among elderly patients with stable coronary artery disease. Acta Cardiol Sin. (2024) 40(1):87–96. 10.6515/ACS.202401_40(1).20230825A 38264075 PMC10801420

[28] Czinege M Ruța F Nyulas V Halațiu V-B Nyulas T Benedek T. Serum albumin concentration and the risk of cardiovascular disease and acute coronary syndrome—a narrative review. J Cardiovasc Emerg. (2025) 11(1):11–9. 10.2478/jce-2024-0024

[29] Liu Y Song J Wang W Zhang K Qi Y Yang J. Association of ejection fraction with mortality and cardiovascular events in patients with coronary artery disease. ESC Heart Fail. (2022) 9(5):3461–8. 10.1002/ehf2.14063 35866195 PMC9715855

[30] Urbanowicz T Michalak M Komosa A Olasińska-Wiśniewska A Filipiak KJ Tykarski A. Predictive value of systemic inflammatory response index (SIRI) for complex coronary artery disease occurrence in patients presenting with angina equivalent symptoms. Cardiol J. (2024) 31(4):583–95. 10.5603/CJ.a2023.0033 37314004 PMC11374332

[31] Guo Z Song S Cheng H Xie C Zhang M Pei M. Combined SHR and SIRI biomarkers predict increased coronary heart disease risk in type 2 diabetes. Biomol Biomed. (2025) 26(3):441–51. 10.17305/bb.2025.13032 40929739 PMC12533829

[32] Zhu L Chen M Lin X. Serum albumin level for prediction of all-cause mortality in acute coronary syndrome patients: a meta-analysis. Biosci Rep. (2020) 40(1):BSR20190881. 10.1042/BSR20190881 31815281 PMC6944666

[33] Collins GS Reitsma JB Altman DG Moons KG. Transparent reporting of a multivariable prediction model for individual prognosis or diagnosis (TRIPOD): the TRIPOD statement. Ann Intern Med. (2015) 162(1):55–63. 10.7326/M14-0697 25560714

