# Supplementary material for: Predictive value of systemic inflammatory response index for coronary artery disease and angiographic severity: a single-center retrospective study
Source: Front Cardiovasc Med. 2026 Jul 29;13:1871311. doi: 10.3389/fcvm.2026.1871311 (PMC13461488; doi:10.3389/fcvm.2026.1871311)
Supplement: Supplementary file 1 [file Table1.docx]

| **Table S1. Clinical characteristics of individuals by cause of hospitalization** | | | | | | | |
| --- | --- | --- | --- | --- | --- | --- | --- |
| Variable | Control (n=94) | S-CAD (n=88) | UA (n=87) | NST-MI (n=83) | ST-MI (n=42) | F/x^2 | p-value |
| Age (years) | 62.23 ± 12.45 | 66.44 ± 9.79 | 64.85 ± 10.34 | 60.64 ± 13.59^b^ | 56.36 ± 12.05^bc^ | 6.775 | <0.001 |
| Gender, male (n, %) | 52 (55.3%) | 63 (71.6%) | 60 (69.0%) | 73 (88.0%) | 37 (88.1%) | 29.447 | <0.001 |
| Diabetes, n (%) | 23 (24.5%) | 28 (31.8%) | 25 (28.7%) | 21 (25.3%) | 8 (19.0%) | 2.944 | 0.567 |
| Hypertension, n (%) | 56 (59.6%) | 64 (72.7%) | 55 (63.2%) | 45 (54.2%) | 22 (52.4%) | 8.268 | 0.082 |
| Hyperlipemia, n (%) | 44 (46.8%) | 20 (22.7%) | 16 (18.4%) | 9 (10.8%) | 2 (4.8%) | 45.812 | <0.001 |
| Weight (kg) | 62.0 (58.5-70.0) | 68.0 (59.8-75.3) | 66.0 (60.0-73.2) | 67.5 (58.5-75.0) | 65.7 (60.0-75.0) | 4.135 | 0.388 |
| Height (cm) | 162.0 (156.0-169.0) | 165.8 (160.0-170.0) | 165.0 (158.8-170.0) | 166.0 (161.1-170.0) | 164.7 (158.3-170.6) | 4.101 | 0.392 |
| BMI (kg/m²) | 24.38 ± 3.07 | 24.84 ± 3.31 | 24.70 ± 3.03 | 24.66 ± 3.83 | 25.07 ± 4.06 | 0.379 | 0.824 |
| Systolic pressure (mmHg) | 134.0 (122.3-146.0) | 145.0 (132.0-153.3)^a^ | 137.0 (127.0-148.0) | 133.0 (122.5-146.0)^b^ | 128.5 (120.3-140.8)^b^ | 17.807 | 0.001 |
| Diastolic pressure (mmHg) | 82.0 (74.3-89.0) | 82.0 (72.0-91.5) | 83.0 (76.5-87.0) | 82.0 (74.0-89.0) | 85.5 (75.0-91.0) | 1.316 | 0.859 |
| LVEF(%) | 67 (65-69) | 66 (64-67) | 66 (63-68) | 63 (60-66)^abc^ | 61 (50-66)^abc^ | 57.192 | <0.001 |
| cTnI (ng/mL) | 0.01 (0.01-0.01) | 0.01 (0.01-0.01) | 0.01 (0.01-0.01) | 0.68 (0.15-5.00)^abc^ | 8.47 (0.83-19.40)^abc^ | 272.058 | <0.001 |
| BNP (pg/mL) | 96.5 (42.5-397.3) | 80.0 (41.8-171.0) | 74.0 (30.0-188.0) | 479.0 (119.5-1055.0)^abc^ | 389.0 (69.0-1504.8)^abc^ | 69.428 | <0.001 |
| WBC (×10⁹/L) | 5.84 (4.75-7.07) | 6.02 (4.84-7.07) | 6.07 (5.09-7.05) | 7.52 (6.29-9.13)^abc^ | 9.96 (8.05-12.00)^abcd^ | 95.123 | <0.001 |
| NE% | 0.60 ± 0.09 | 0.63 ± 0.09 | 0.62 ± 0.08 | 0.69 ± 0.09^abc^ | 0.75 ± 0.10^abcd^ | 27.488 | <0.001 |
| LY% | 0.30 ± 0.08 | 0.27 ± 0.08 | 0.28 ± 0.08 | 0.23 ± 0.08^abc^ | 0.18 ± 0.08^abc^ | 25.427 | <0.001 |
| MO% | 0.07 (0.06-0.09) | 0.07 (0.06-0.08) | 0.07 (0.06-0.09) | 0.07 (0.06-0.08) | 0.06 (0.05-0.08) | 6.39 | 0.172 |
| NE (×10⁹/L) | 3.40 (2.85-4.28) | 3.52 (2.88-4.83) | 3.68 (2.92-4.40) | 4.87 (4.16-6.27)^abc^ | 7.31 (6.20-9.07)^abcd^ | 113.26 | <0.001 |
| LY (×10⁹/L) | 1.73 (1.46-2.18) | 1.55 (1.22-1.98) | 1.66 (1.33-2.05) | 1.59 (1.29-1.96) | 1.57 (1.11-2.30) | 5.858 | 0.210 |
| MO (×10⁹/L) | 0.40 (0.31-0.53) | 0.41 (0.33-0.49) | 0.43 (0.34-0.53) | 0.50 (0.43-0.66)^abc^ | 0.57 (0.41-0.78)^abc^ | 38.89 | <0.001 |
| PLT (×10⁹/L) | 213.5 (185.3-244.0) | 206.5 (172.3-237.0) | 204.0 (180.5-237.5) | 213.0 (178.0-249.0) | 233.0 (200.0-261.0) | 8.903 | 0.064 |
| INR | 0.97 (0.94-1.02) | 0.98 (0.93-1.02) | 0.99 (0.95-1.04) | 1.01 (0.97-1.07)^ab^ | 1.01 (0.98-1.07)^ab^ | 19.092 | <0.001 |
| APTT (s) | 34.95 (33.4-37.00) | 35.40 (33.98-36.95) | 35.3 (33.3-38.00) | 37.50 (34.50-41.5)^abc^ | 38.05 (34.52-45.12)^abc^ | 27.788 | <0.001 |
| FIB (g/L) | 3.04 (2.60-3.50) | 3.23 (2.79-3.65) | 3.04 (2.70-3.44) | 3.45 (2.80-4.01)^a^ | 3.23 (2.87-4.45)^a^ | 18.61 | <0.001 |
| DD (mg/L) | 0.23 (0.22-0.34) | 0.24 (0.22-0.39) | 0.22 (0.22-0.32) | 0.26 (0.22-0.45) | 0.28 (0.22-0.44) | 7.34 | 0.119 |
| ALT (U/L) | 21.0 (16.0-30.8) | 22.0 (16.5-31.0) | 20.0 (15.0-33.0) | 24.0 (18.0-34.5) | 32.0 (23.0-43.0)^abc^ | 12.771 | 0.012 |
| AST (U/L) | 22.5 (20.0-26.0) | 22.0 (18.0-28.3) | 22.0 (17.0-26.0) | 31.0 (20.0-51.5)^abc^ | 47.5 (29.3-77.8)^abcd^ | 67.63 | <0.001 |
| ALP (U/L) | 76.5 (66.3-101.0) | 89.5 (71.5-108.0) | 83.0 (70.0-101.5) | 92.0 (77.5-113.5)^a^ | 89.5 (74.0-141.8) | 11.777 | 0.019 |
| GGT (U/L) | 28.0 (19.0-41.0) | 30.0 (19.0-51.5) | 26.0 (15.0-49.0) | 38.0 (22.0-66.0)^c^ | 40.5 (21.3-71.8) | 11.917 | 0.018 |
| Albumin (g/L) | 43.4 (40.8-45.0) | 42.3 (40.0-44.8) | 42.5 (40.6-44.2) | 39.6 (37.0-43.2)^abc^ | 39.4 (37.8-41.3)^abc^ | 41.449 | <0.001 |
| **Table S1. Clinical characteristics of individuals by cause of hospitalization (Continued)** | | | | | | | |
| Variable | Control (n=94) | S-CAD (n=88) | UA (n=87) | NST-MI (n=83) | ST-MI (n=42) | F/x^2 | p-value |
| Total bilirubin (μmol/L) | 11.1 (8.0-15.9) | 11.7 (9.4-16.0) | 11.6 (9.0-15.3) | 13.0 (9.6-16.3) | 12.9 (8.7-17.5) | 4.027 | 0.402 |
| Direct bilirubin (μmol/L) | 2.2 (1.6-3.0) | 2.5 (1.8-3.4) | 2.4 (1.8-3.6) | 2.6 (1.9-4.0) | 2.7 (1.9-3.7) | 6.417 | 0.170 |
| Indirect bilirubin (μmol/L) | 9.1 (7.1-12.7) | 9.6 (7.9-12.5) | 9.1 (7.3-12.6) | 9.7 (8.0-11.85) | 9.6 (8.5-13.6) | 0.606 | 0.606 |
| Glu (mmol/L) | 6.11 (5.24-8.01) | 6.74 (5.21-8.89) | 6.62 (5.37-8.08) | 6.71 (5.55-9.14) | 6.70 (5.74-9.27) | 4.069 | 0.397 |
| HBA1C (%) | 5.89 (5.52-6.38) | 6.04 (5.48-7.00) | 5.82 (5.49-6.60) | 5.87 (5.55-7.08) | 5.67 (5.5-6.32) | 1.623 | 0.805 |
| Cr (μmol/L) | 66.1 (55.3-77.0) | 74.8 (62.1-90.5)^a^ | 71.0 (57.7-80.3) | 69.0 (61.0-79.6) | 73.5 (65.7-84.5)^a^ | 15.309 | 0.004 |
| Creatine kinase (U/L) | 91.0 (68.0-127.5) | 95.5 (64.0-143.0) | 94.0 (68.0-136.5) | 160 (87.0-403.5)^abc^ | 503.5 (146.8-982.0)^abcd^ | 76.008 | <0.001 |
| LDH (U/L) | 178.0 (152.5-194.9) | 169.0 (151.0-194.3) | 168.0 (147.3-193.0) | 197.0 (166.5-248.0)^abc^ | 246.0 (199.3-386.0)^abcd^ | 54.001 | <0.001 |
| Triglyceride (mmol/L) | 1.77 (1.17-2.44) | 1.83 (1.28-2.90) | 1.81 (1.11-2.71) | 1.68 (1.25-2.40) | 1.53 (1.04-2.82) | 3.647 | 0.456 |
| Total cholesterol (mmol/L) | 4.87 (4.15-5.63) | 4.59 (3.88-5.61) | 4.70 (4.08-5.61) | 4.84 (3.90-5.70) | 5.21 (4.39-5.97) | 5.497 | 0.240 |
| HDL (mmol/L) | 1.21 (1.04-1.36) | 1.14 (1.00-1.37) | 1.13 (0.94-1.34) | 1.02 (0.83-1.19)^ab^ | 1.03 (0.89-1.23)^a^ | 26.05 | <0.001 |
| LDL-c (mmol/L) | 2.90 (2.50-3.60) | 2.88 (2.19-3.50) | 2.87 (2.40-3.55) | 3.09 (2.62-3.72) | 3.42 (2.80-4.33)^abc^ | 16.375 | 0.003 |
| apoA (g/L) | 1.25 (1.15-1.42) | 1.22 (1.09-1.30) | 1.20 (1.09-1.31) | 1.10 (0.99-1.18)^abc^ | 1.08 (1.02-1.18)^abc^ | 46.44 | <0.001 |
| apoB (g/L) | 0.94 (0.78-1.13) | 0.90 (0.78-1.10) | 0.93 (0.78-1.11) | 0.98 (0.80-1.23) | 1.14 (0.87-1.34)^bc^ | 10.642 | 0.031 |
| Cholinesterase (U/L) | 8042.5 (7039.3-9857.5) | 8423.0 (7351.0-9348.2) | 8386.0 (7202.7-9344.0) | 8121.0 (6416.0-9218.5) | 7797.0 (6935.6-9450.8) | 2.55 | 0.636 |
| Kalium (mmol/L) | 3.94 (3.75-4.13) | 3.87 (3.64-4.09) | 3.83 (3.62-4.05) | 3.88 (3.71-4.08) | 3.88 (3.64-4.05) | 4.719 | 0.317 |
| Gensini score | 0.0 (0.0-3.4 ) | 21.5 (10.0-48.3 )^a^ | 17.0 (9.5-27.5 )^a^ | 42.0 (32.0-58.5 )^abc^ | 55.0 (42.3-82.0 )^abc^ | 243.053 | <0.001 |
| SIRI | 0.78 (0.60-1.13) | 0.96 (0.64-1.49) | 0.87 (0.68-1.34) | 1.59 (1.06-2.37)^abc^ | 2.62 (1.57-3.62)^abc^ | 101.914 | <0.001 |

Note. Clinical characteristics of patients stratified by cause of hospitalization Continuous variables are presented as mean ± standard deviation ($\bar{x}$ ± s) for continuous variables with normal distribution and as median with 25%-75% interquartile range, M(Q3-Q1) for continuous variables with non-normal distribution; categorical variables as n (%).

*p-values from the parameters were compared between patients with different groups using Kruskal–Wallis analysis of variance with multiple comparisons performed using Dunn’s test in case of significant differences with statistical significance across all five clinical subgroups: non-coronary artery disease (Non-CHD), stable coronary artery disease (S-CAD), unstable angina (UA), non–ST-segment elevation myocardial infarction (NSTEMI), and ST-segment elevation myocardial infarction (STEMI). ^a^*p* < 0.05 vs control; ^b^*p* < 0.05 vs S-CHD; ^c^*p* < 0.05 vs UA; ^d^*p* < 0.05 vs NSTEMI.

**Table S2. ROC analysis of six inflammation-based indices for identifying angiographic CAD.**

| **Inflammatory index** | **AUC (95% CI)** | **P value** | **Sensitivity** | **Specificity** |
| --- | --- | --- | --- | --- |
| SIRI | 0.684 (0.625-0.744) | <0.001 | 0.487 | 0.819 |
| NLR | 0.679 (0.618-0.739) | <0.001 | 0.723 | 0.543 |
| AISI | 0.657 (0.596-0.718) | <0.001 | 0.517 | 0.745 |
| SII | 0.647 (0.586-0.709) | <0.001 | 0.660 | 0.574 |
| MLR | 0.635 (0.574-0.696) | <0.001 | 0.613 | 0.670 |
| PLR | 0.543 (0.478-0.608) | 0.193 | 0.243 | 0.872 |

Note. AUC values were tested against the null value of 0.5. Sensitivity and specificity were determined using the Youden index. CAD, coronary artery disease; AUC, area under the receiver operating characteristic curve; CI, confidence interval; SIRI, systemic inflammatory response index; NLR, neutrophil-to-lymphocyte ratio; PLR, platelet-to-lymphocyte ratio; MLR, monocyte-to-lymphocyte ratio; SII, systemic immune-inflammation index; AISI, aggregate index of systemic inflammation.

**Table S3. Pairwise DeLong comparisons between SIRI and other inflammation-based indices.**

| **Comparison** | **AUC of SIRI** | **AUC of comparator** | **DeLong P value** |
| --- | --- | --- | --- |
| SIRI vs NLR | 0.684 | 0.679 | 0.791 |
| SIRI vs PLR | 0.684 | 0.543 | <0.001 |
| SIRI vs MLR | 0.684 | 0.635 | 0.01 |
| SIRI vs SII | 0.684 | 0.647 | 0.152 |
| SIRI vs AISI | 0.684 | 0.657 | 0.069 |

Note. Pairwise comparisons were performed using DeLong tests for correlated ROC curves. P values indicate whether the AUC of SIRI differed significantly from that of the corresponding inflammatory index.

**Table S4. Stability analysis of multivariable CAD models using different SIRI scaling methods.**

| **SIRI scaling method** | **Model** | **AUC (95% CI)** | **AIC** | **BIC** | **Calibration intercept** | **Calibration slope** | **Brier score** |
| --- | --- | --- | --- | --- | --- | --- | --- |
| Raw SIRI | albumin+LVEF+SIRI | 0.740 (0.682-0.797) | 394.759 | 410.665 | 0.000 | 1.000 | 0.161 |
| log10(SIRI) | albumin+LVEF+log10(SIRI) | 0.740 (0.683-0.796) | 392.096 | 408.001 | -0.000 | 1.000 | 0.160 |
| SIRI quartiles | albumin+LVEF+SIRI quartiles | 0.742 (0.687-0.798) | 391.588 | 415.446 | -0.000 | 1.000 | 0.158 |

Note. Each model included albumin and LVEF, with SIRI entered as a raw continuous variable, log10-transformed variable, or quartile-based variable. Calibration metrics were derived from internal validation. AIC, Akaike information criterion; BIC, Bayesian information criterion; LVEF, left ventricular ejection fraction.

**Table S5. ROC analysis of SIRI scaling methods and corresponding multivariable models for CAD prediction.**

| **Predictor or model** | **AUC (95% CI)** | **P value** | **Sensitivity** | **Specificity** |
| --- | --- | --- | --- | --- |
| SIRI | 0.684 (0.625-0.744) | <0.001 | 0.487 | 0.819 |
| log10(SIRI) | 0.684 (0.625-0.744) | <0.001 | 0.487 | 0.819 |
| SIRI quartiles | 0.680 (0.623-0.737) | <0.001 | 0.563 | 0.713 |
| albumin+LVEF+SIRI | 0.740 (0.682-0.797) | <0.001 | 0.697 | 0.745 |
| albumin+LVEF+log10(SIRI) | 0.740 (0.683-0.796) | <0.001 | 0.657 | 0.755 |
| albumin+LVEF+SIRI quartiles | 0.742 (0.687-0.798) | <0.001 | 0.673 | 0.745 |

Note. This table compares untransformed SIRI values, log_10_SIRI, SIRI quartiles, and the corresponding albumin + LVEF-based multivariable models. The analysis was performed to evaluate whether model discrimination was affected by continuous modeling, logarithmic transformation, or categorical representation of SIRI. Sensitivity and specificity were determined using the Youden index. P values refer to ROC tests against the null AUC of 0.5.

**Table S6. Subgroup ROC analyses across CAD phenotypes compared with the non-CAD control group.**

| **Subgroup** | **Events/controls, n** | **Predictor or model** | **AUC (95% CI)** | **P value** | **Sensitivity** | **Specificity** |
| --- | --- | --- | --- | --- | --- | --- |
| Stable CAD | 88/94 | Gensini score | 0.966 (0.945-0.986) | <0.001 | 0.818 | 0.957 |
| Stable CAD | 88/94 | albumin+LVEF+SIRI | 0.642 (0.561-0.723) | <0.001 | 0.750 | 0.532 |
| Stable CAD | 88/94 | albumin+LVEF | 0.613 (0.531-0.695) | 0.009 | 0.864 | 0.340 |
| Stable CAD | 88/94 | LVEF | 0.589 (0.507-0.672) | 0.037 | 0.750 | 0.415 |
| Stable CAD | 88/94 | SIRI | 0.584 (0.500-0.667) | 0.052 | 0.682 | 0.511 |
| Stable CAD | 88/94 | albumin | 0.570 (0.486-0.653) | 0.104 | 0.693 | 0.489 |
| Stable CAD | 88/94 | cTnI | 0.490 (0.440-0.539) | 0.684 | 1.000 | 0.000 |
| UA | 87/94 | Gensini score | 0.962 (0.940-0.984) | <0.001 | 0.828 | 0.957 |
| UA | 87/94 | albumin+LVEF+SIRI | 0.612 (0.529-0.695) | 0.009 | 0.644 | 0.617 |
| UA | 87/94 | albumin+LVEF | 0.609 (0.526-0.692) | 0.011 | 0.621 | 0.617 |
| UA | 87/94 | LVEF | 0.572 (0.488-0.655) | 0.094 | 0.448 | 0.681 |
| UA | 87/94 | SIRI | 0.567 (0.483-0.651) | 0.119 | 0.759 | 0.372 |
| UA | 87/94 | albumin | 0.565 (0.481-0.649) | 0.133 | 0.609 | 0.532 |
| UA | 87/94 | cTnI | 0.509 (0.457-0.562) | 0.735 | 0.161 | 0.862 |
| NSTEMI | 83/94 | Gensini score | 0.996 (0.990-1.000) | <0.001 | 0.964 | 1.000 |
| NSTEMI | 83/94 | cTnI | 0.939 (0.901-0.978) | <0.001 | 0.988 | 0.862 |
| NSTEMI | 83/94 | albumin+LVEF+SIRI | 0.855 (0.800-0.911) | <0.001 | 0.880 | 0.745 |
| NSTEMI | 83/94 | SIRI | 0.809 (0.745-0.872) | <0.001 | 0.687 | 0.798 |
| NSTEMI | 83/94 | albumin+LVEF | 0.788 (0.720-0.856) | <0.001 | 0.759 | 0.745 |
| NSTEMI | 83/94 | LVEF | 0.749 (0.677-0.822) | <0.001 | 0.735 | 0.681 |
| NSTEMI | 83/94 | albumin | 0.704 (0.628-0.781) | <0.001 | 0.542 | 0.840 |
| STEMI | 42/94 | Gensini score | 1.000 (1.000-1.000) | <0.001 | 1.000 | 1.000 |
| STEMI | 42/94 | cTnI | 0.961 (0.928-0.994) | <0.001 | 0.952 | 0.926 |
| STEMI | 42/94 | albumin+LVEF+SIRI | 0.947 (0.914-0.980) | <0.001 | 0.976 | 0.830 |
| STEMI | 42/94 | SIRI | 0.891 (0.834-0.948) | <0.001 | 0.738 | 0.926 |
| STEMI | 42/94 | albumin+LVEF | 0.848 (0.776-0.919) | <0.001 | 0.810 | 0.755 |
| STEMI | 42/94 | LVEF | 0.796 (0.709-0.883) | <0.001 | 0.619 | 0.840 |
| STEMI | 42/94 | albumin | 0.764 (0.675-0.852) | <0.001 | 0.762 | 0.691 |

Note. For each subgroup, the indicated CAD phenotype was compared with the non-CAD control group. Gensini score was included as an angiographic comparator and was not used as a predictor in the primary pre-angiographic nomogram. UA, unstable angina; NSTEMI, non-ST-segment elevation myocardial infarction; STEMI, ST-segment elevation myocardial infarction; cTnI, cardiac troponin I.
